# Efficacy and safety of Gemogenovatucel-T (Vigil) immunotherapy for advanced ovarian carcinoma: A systematic review and meta-analysis of randomized controlled trials

**DOI:** 10.3389/fonc.2022.945867

**Published:** 2022-10-21

**Authors:** Yixin Zhang, Li Zhang, Yuli Zhao, Sen Wang, Li Feng

**Affiliations:** ^1^ Department of Medical Ultrasound, The First Affiliated Hospital of Shandong First Medical University & Shandong Provincial Qianfoshan Hospital, Shandong Medicine and Health Key Laboratory of Abdominal Medical Imaging, Jinan, China; ^2^ Department of Medical Ultrasound, Shandong Provincial Qianfoshan Hospital, Shandong First Medical University, Jinan, China

**Keywords:** ovarian carcinoma, Gemogenovatucel-T, Vigil, meta-analysis, immunotherapy

## Abstract

In recent years, many clinical trials have shown the safety and efficacy of Gemogenovatucel-T (Vigil) in the treatment of advanced OC patients. The purpose of this study was to explore the safety and efficacy of Gemogenovatucel-T (Vigil) in the first-line maintenance of advanced ovarian carcinoma based on the randomized controlled trials (RCTs). Randomized controlled trials (RCTs) on Gemogenovatucel-T (Vigil) immunotherapy for advanced ovarian carcinoma were searched in PubMed, Embase, Cochrane Library and Web of Science up to December 31, 2021. The following study characteristics were investigated: baseline study characteristics, overall survival, recurrence free survival, recurrence free survival median time, and complication. A total of 36 articles were obtained, and seven suitable RCTs with a total sample size of 322 patients were eventually included in this meta-analysis. Overall survival (OS): from time of randomization: HR=0.48 (95% CI: 0.32 to 0.72), Z=3.55, P<0.001; from time of tissue procurement: HR=0.51 (95% *CI*: 0.33 to 0.78), *Z*=3.07, *P*=0.002. Recurrence free survival (RFS): from time of randomization: HR=0.43 (95% *CI*: 0.30 to 0.62), *Z*=4.61, *P*<0.001; from time of tissue procurement: HR=0.45 (95% *CI*: 0.31 to 0.65), *Z*=4.26, *P*<0.001. RFS median time: from time of randomization: HR=1.57 (95% *CI*: 1.16 to 2.11), *Z*=2.95, *P*=0.003; from time of tissue procurement: HR=2.16 (95% *CI*: 1.12 to 4.17), *Z*=2.29, *P*=0.022. This study found that Gemogenovatucel-T (Vigil) immunotherapy provided significant OS and RFS benefits, particularly in advanced OC patients with BRCA wild type. At the same time, treatment with the Gemogenovatucel-T (Vigil) is safer than other treatment modalities and does not have any toxic effects.

**Systematic Review Registration:**

https://www.crd.york.ac.uk/prospero/, identifier (CRD42022300367).

## 1 Introduction

Ovarian carcinoma (OC) ranks as the second major cause of gynaecological cancer mortality among females worldwide (1). It is still a challenging and difficult disease to treat, partly due to advanced ovarian carcinoma (2). Globally, an incidence of around 239,000 new cases and 152,000 deaths are reported in 2020 (3). The 5-year survival rate for patients with newly diagnosed advanced surgical resectable disease receiving optimal standard of care treatment, comprising tumor reduction surgery followed by neoadjuvant or adjacent chemotherapy with paclitaxel and carboplatin and with or without bevacizumab, was only 48% (4, 5). Survival rates for stage IV patients are even worse, with a 5-year survival rate under 20% (6). Although the majority of patients obtain complete remission by both methods, recurrence occurs in almost 75% of patients within two years (4).

For nearly 20 years, platinum-based chemotherapy in combination with paclitaxel has been the standard treatment for ovarian carcinoma patients (7). However, it was recently shown in a meta-analysis among ovarian carcinoma patients that the inefficient repair mechanisms that result may be mutated (-m) tumors predicted an improved response rate to platinum-based chemotherapy compared with BRCA wild type (BRCA-wt) tumors (8). Poly (ADP-ribose) polymerase (PARP) inhibitors and bevacizumab have been used in recent years to strive to improve the prognosis of first line treated ovarian carcinoma by providing maintenance therapy after patients had achieved a complete response (9–11). On the other hand, several studies have shown that they benefit preferentially in patients with homologous recombination deficiency (HRD) cancers and less in those with homologous recombination proficient (HRP) ovarian carcinoma patients (12, 13). Additionally, when administered in high doses, several PARP inhibitors and bevacizumab may cause toxic drug-related effects (14). Therefore, more comprehensive treatment modalities or medications that can increase the responsiveness and survival of patients with advanced OC are required.

Gemogenovatucel-T (Vigil) is a novel ovarian carcinoma maintenance therapy that is an autologous tumor cell vaccine produced from malignant tissue collected during debulking surgery (15). Gemogenovatucel-T (Vigil) constructs comprising a dual-function short hairpin RNA construction and a plasmid carrying the human immunostimulatory GMCSF gene, as well as the downstream targets (TGF β (beta)1 and TGF β (beta)2), are selectively knocked down by the proprotein convertase furin (16–18). In addition, exogenous GM-CSF production promotes immune function and enhanced antigen expression (19). Vigils versus Placebo maintenance treatment in patients with newly diagnosed stage IIIB-IV resectable ovarian carcinoma with BRCA-wt demonstrated OS and RFS benefits in the phase IIb trial (VITAL study) (20). It was later shown that when compared to the BRCA-m or BRCA HRD group, the HRP group was the most sensitive to Vigil maintenance therapy (21). Meanwhile, some studies revealed Gemogenovatucel-T (Vigil) immunotherapy to be completely safe (17). A recent three-year long-term follow-up study confirms the long-term safety of Vigil treatment for patients with advanced OC (22).

Even though multiple clinical trials have demonstrated the efficacy and safety of Gemogenovatucel-T (Vigil) in the treatment of advanced OC patients, particularly those with BRCA-wt status, Evidence based on Evidence-based medicine is still lacking. Therefore, we conducted this meta-analysis based on randomized controlled trials (RCTs) to explore the safety and efficacy of Gemogenovatucel-T (Vigil) in the front-line upkeep of advanced OC.

## 2 Materials and methods

This systematic review program was registered on PROSPERO (ID=CRD42022300367). This meta-analysis review was prepared in accordance with the Preferred Reporting Items for Systematic Reviews and Meta-Analyses (PRISMA) statement (23) and included all studies based on the randomized controlled trials (RCTs) with no restrictions on the year of publication.

### 2.1 Sources of data and searching strategy

The search period was from the establishment of each database through December 31, 2021, with no constraints on the year of publication. We searched PubMed, Embase, the Cochrane Library, and Web of Science for all published randomized controlled trials on Gemogenovatucel-T (Vigil) immunotherapy for advanced ovarian carcinoma. The retrieval strategy was based on a topic phrase plus free words approach, and supplemental online materials provides detailed search strategies for each database. **Supplementary Tables S2–S5** shows the search strategy in the databases above.

### 2.2 Literature inclusion and exclusion criteria

Inclusion of studies if they comply with all of the below inclusion criteria: (a) The intervention was Gemogenovatucel-T (Vigil) immunotherapy; (b) the trial participants were ovarian carcinoma patients; and (c) the literature was in English.

Studies were excluded if any the following exclusion criteria were present: (a) *in vitro* experiments (animal experiments), reviews, conference abstracts, letters, retrospective studies, guidelines, case reports, pathological mechanisms, and so on; (b) studies on non-ovarian carcinoma patients; (c) literature with a sample size of <20 patients; (d) literature in other languages; (e) the original literature cannot be obtained; (f) duplicate documents, secondary analysis counts duplicate documents; and (g) literature of low quality or with serious errors in design.

### 2.3 Literature screening and data extraction

Two researchers (YXZ and YLZ) independently identified duplicates and screen studies using the EndNote X9.3.3 software for Macintosh. After excluding duplicates, the remaining literature was screened using titles and abstracts to remove studies on unrelated topics. This study was then checked against the full text to determine which publications should be included.

Data were extracted from all eligible studies by two investigators using a standardized data acquisition form. The following data were extracted for each of the studies included in the meta-analysis: general information for inclusion in the literature (first author, publication year, disease setting, etc.), patient information (sample size, ECGO performance status, BRCA status of included patients, etc.), intervention measures, course of treatment and endpoints indicators. Prognosis if graphed as a Kaplan-Meier curve alone [in only one case (24)], then the data was digitized and extracted using the software Engauge Digitizer 12.1 for Macintosh (https://markummitchell.github.io/engauge-digitizer/).

The above literature screening and data extraction were undertaken by two investigators (YXZ and YLZ) independently. The results would be cross-checked by both researchers after the process was completed. Any disagreements were resolved by discussion and consensus with the third researcher (LF). The third researcher would assist in decision-making.

### 2.4 Endpoints indicators

The primary endpoints for patients in this meta-analysis were OS, RFS, and RFS median time, whereas the secondary endpoint was the characterization of the patient’s severe adverse reactions (G3/G4 toxicity). Severe toxicity features included some gastrointestinal disorders, hypertension, thrombocytopenia and neutropenia.

The PFS of the patient was not deemed to be an endpoint in this meta-analysis as the data could only be used for one study (25).

### 2.5 Quality evaluation

Two independent researchers identified and selected studies based on the inclusion and exclusion criteria using the Cochrane Collaboration Risk of Bias Tool (CCRBT) (26). The CCRBT comprises six items particularly designed to assess the risk of bias in the following six domains (1): Selection bias, including allocation concealment and random sequence generation (2); Performance bias (Blinding of participants and personnel) (3); Detection bias (Blinding of outcome assessment) (4); Attrition bias (Incomplete outcome data) (5); Reporting bias (Selective reporting) (6); Other bias (Anything else, ideally prespecified). The risk of bias was divided into “high risk”, “low risk” or “unclear risk” of bias for each category. Any disagreements in the quality assessment results were resolved by discussion with co-authors, and the third researcher (LF) eventually assisted in adjudication.

### 2.6 Statistical analysis

Stata 15.0 (StataCorp., College Station, TX, USA) software was used for statistical analysis, and the outcome indicators in this meta-analysis were HR for OS, RFS, and the median time for RFS, as well as their confidence intervals (CI). The index of inconsistency (I^2^) was used to reflect heterogeneity between studies. When I^2^ < 50%, the combined effect size was used in a fixed-effects model, however when I^2^≥50%, heterogeneity was considered to be substantial, at which point a random-effects model was used. Due to the small number of studies included in this meta-analysis, an analysis of publication bias was not performed. p<0.05 was considered a statistical difference.

## 3 Results

### 3.1 Results of literature search

The search of the literature yielded a total of 36 studies. Due to duplication, 16 studies (44.4%) were eliminated. After evaluating the title and abstract, twelve publications (33.3%) were removed, two of which were editorial type articles and the other 10 were all conference abstracts. One study (2.8%) was subsequently excluded from the full text assessment owing to phase II trial of maintenance vigil for advanced ovarian carcinoma is still ongoing (27). Ultimately, we included seven randomized controlled trials in this meta-analysis. It is worth noting that in the seven studies that were eventually included, the vaccine manufacturer and the trial sponsor was always Gradalis, which is a biotech company developing this tumor vaccine.

**Figure 1:**
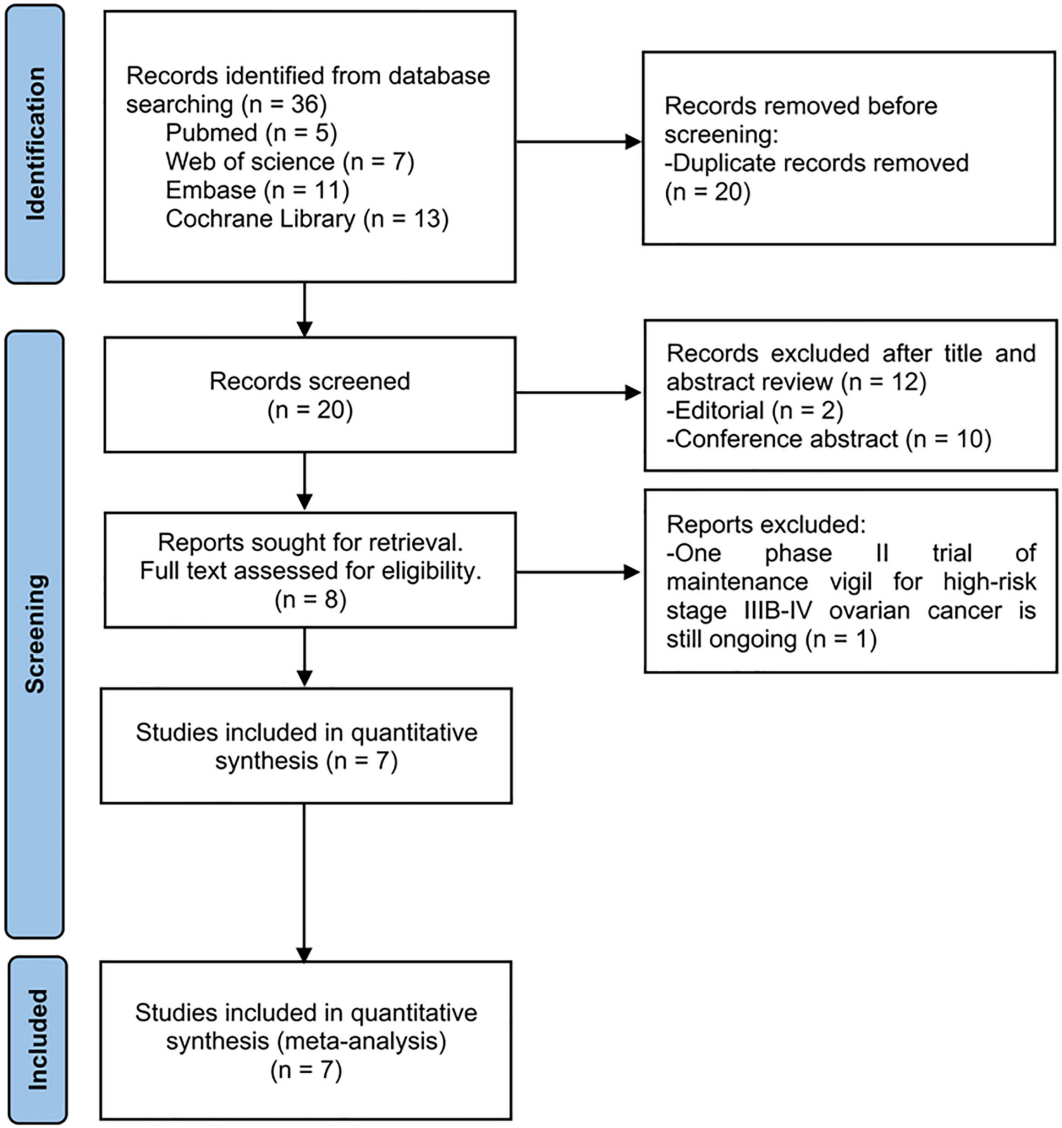
Flow chart of literature screening process.

A PRISMA flowchart summarizing the evidence acquisition process is shown in **Figure 1**. The flowchart sets out a series of processes for literature screening (identified, screened, included, and excluded) and the reasons for exclusion.

### 3.2 General features of the included literature

Inclusion of studies published between 2012 and 2021 with a total of 322 patients, comprising 187 in the experimental group (Gemogenovatucel-T (Vigil) immunotherapy) and 135 in the control group (other therapy), the dosage and frequency of the administration were not exactly the same in all trials, see **Tables 1**; **S1** for main characteristics of the selected studies.

**Table 1:** General features of the included literature.

First Author	Year of publication	NCT number	Disease setting	Study type	ECGO performance status	Total N. of pts	N. of experiment pts	N. of control pts	N. of BRCAm pts	N. of BRCAwt pts	N. of BRCA unknown status	Experimental arm (n. of pts assigned)	Control arm (n. of pts assigned)	Course of treatment	Outcome indicators
Rodney P. Rocconi(21)	2021	NCT02346747	Stage III/IV high grade serious, endometroid or clear cell ovarian cancer	Phase 2b, double blind, RCT	0、1	45	25	20	0	45	NA	1×10^7^cells/injection of Vigil/month (25)	Placebo/month (20)	a minimum of 4 and maximum of 12 doses	RFS, OS
Rodney P. Rocconi(2)	2021 (1)	NCT01061840 NCT01309230	Recurrent ovarian cancer patients	RCT	0、1	33	21	12	NA	NA	NA	Vigil at 1×10^7^ or 2.5×10^7^ cells/injection (21)	NanoString^®^ (12)	NA	OS
Adam Walter(28)	2021	NCT02346747	High-grade papillary serous, clear cell, endometrioid ovarian, fallopian tube or primary peritoneal cancer	Phase 2b, double blind, RCT	0、1	45	25	20	0	45	NA	1x10e^7^cells/injection of Vigil/month (25)	Placebo/month (20)	a minimum of 4 and maximum of 12 doses	RFS, OS
JonathanOh(15)	2016	NCT01551745	Stages III/IV serous/endometrioid ovarian/fallopian cancer or primary peritoneal cancer	Phase II open-label trial, RCT	NA	42	31	11	NA	NA	NA	1.0×10^7^cells/intradermal injection of Vigil/month (31)	SOC (standard of care maintenance therapy) (11)	a minimum of 4 and maximum of 12 doses	RFS
NeilSenzer(17)	2012	NCT01505153	A histologically confirmed advanced or metastatic nondurable solid tumor following completion of ≥1 disease appropriate standard of care therapy and recovery from all treatment-related toxicities to ≤ grade 1 (except alopecia)	Phase I, nonrandomized, open-label trial	0、1	45	27	18	NA	NA	NA	1 × 10^7^ cells/injection (cohort 1) or 2.5 × 10^7^ cells/injection (Cohort 2) of FANG (27)	no FANG (18)	a minimum of 5 monthly injections, maximum of 12 intradermal injections	OS
Rodney P. Rocconi(20)	2020	NCT02346747	Stage III or IV high-grade serous, endometrioid, or clear cell ovarian cancer in clinical completeresponse after a combination of surgery and five to eight cycles of chemotherapy involving carboplatin and paclitaxel	Phase 2b, double blind, RCT	0、1	91	47	44	24	67	NA	1×10^7^ cells per intradermal injection of Gemogenovatucel-T/month (47)	Placebo/month (44)	a minimum of four and up to 12 doses (within 8 weeks after last chemotherapy)	RFS, OS
Rodney P. Rocconi(25)	2021 (2)	NCT03073525	Stages III/IV HGSC (high-grade serous carcinoma)	phase 1,2-part, open-label trial, RCT	0、1	21	11	10	7	13	1	received Vigil (1 × 10e^6^–10e^7^ cells/dose intradermally)/three weeks (11)	atezolizumab (1200 mg/dose intravenous infusion)/three weeks (10)	up to 12 doses	PFS, OS

NA, not available.

There are some differences in doses, which is a limitation that is difficult to avoid in the meta-analysis of RCTs. In our study, there was only very small heterogeneity in OS and PFS, which means that different doses did not have a very large effect on them. However, may have some effect in RFS. Due to the limited number of included studies, further exploration is needed in the future.

### 3.3 Quality evaluation of the included literature

The Cochrane Risk of Bias Assessment Tool was used to assess the risk of bias in included studies. The risk rating for each step-in selection bias, performance bias, attrition bias, reporting bias and other bias are shown in **Table 2**, and the graphs are presented *via* Revman 5.4 software. As the results show, there is no high risk of bias in selective reporting (**Figures S1**, **S2**).

**Table 2:** Cochrane Collaboration’s tool for evaluation risk of bias in included studies.

Author	Year	V1	V2	V3	V4	V5	V6	V7
Rodney P. Rocconi	2021	Low	Low	Low	Low	Low	Low	Low
Rodney P. Rocconi	2021 (1)	Unclear	Unclear	Unclear	Low	Low	Unclear	Low
Adam Walter	2021	Low	Low	Low	Low	Low	Low	Low
Jonathan Oh	2016	Low	Unclear	Low	Low	Low	Low	Low
Neil Senzer	2012	Unclear	Unclear	Low	Low	Low	Unclear	Low
Rodney P. Rocconi	2020	Low	Low	Low	Low	Low	Low	Low
Rodney P. Rocconi	2021 (2)	Low	Low	Low	Low	Low	Low	Low

V1, random sequence generation; V2, allocation concealment; V3, blinding of participants and personnel; V4, blinding of outcome assessment; V5, incomplete outcome data; V6, selective reporting; V7, other bias.

### 3.4 Meta-analysis outcomes

#### 3.4.1 Overall survival

**Figure 2:**
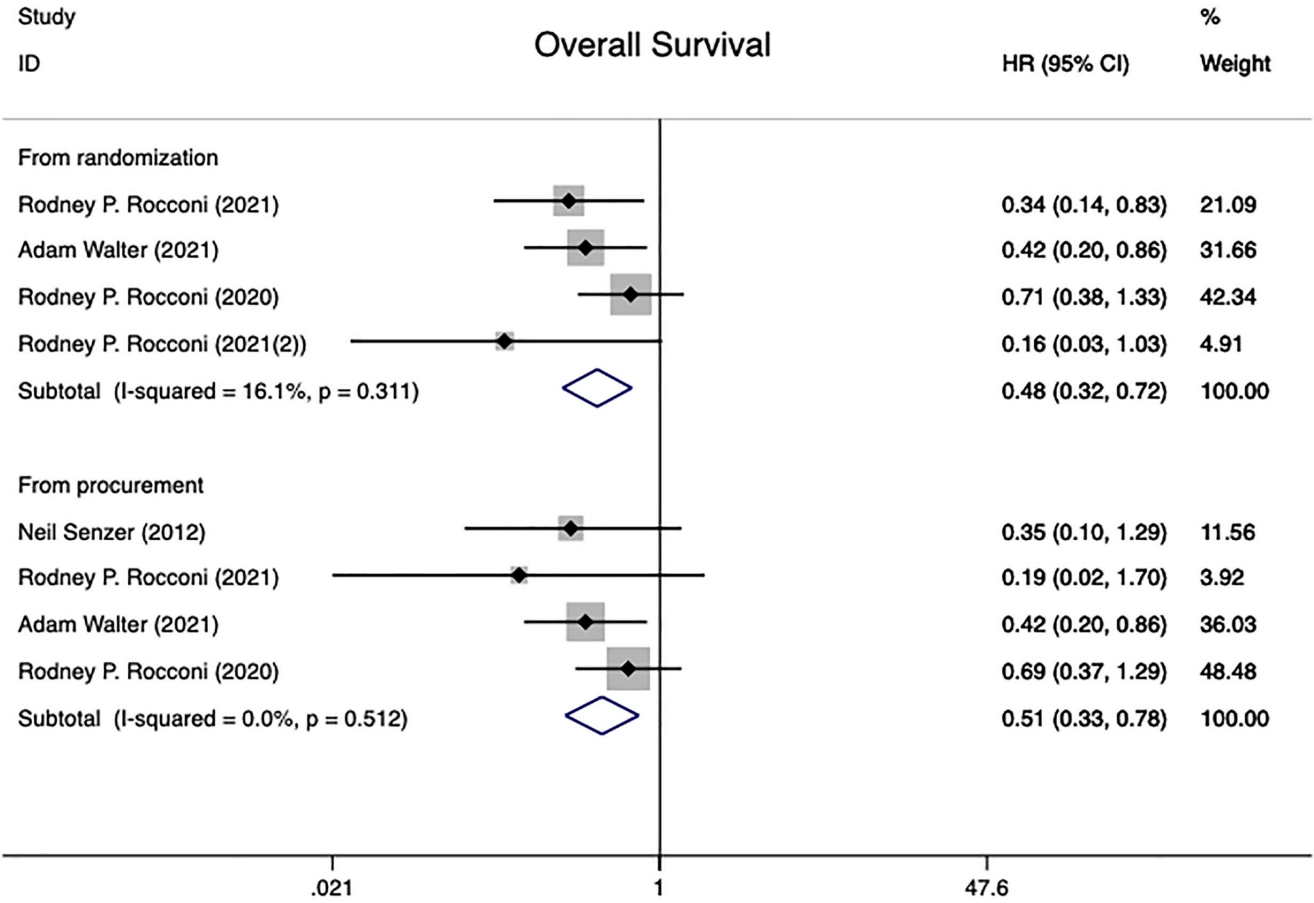
Overall survival in advanced OC patients at randomization and from time of tissue procurement time by using a fixed-effect model. OC, ovarian carcinoma; CI, confidence interval; HR, hazard ratio.

This research comprised 202 patients who participated in four different randomized controlled trials (20, 21, 25, 28), all of which reported the overall survival HR. All patients underwent analysis of the effect of treatment in the time of randomization versus from the time of tissue procurement groups. The time of randomization group had low between-study heterogeneity (I^2 =^ 16.1%, P=0.311), whereas the time of tissue procurement group had no heterogeneity in overall survival (I^2 =^ 0.0%, P=0.512).Using a fixed effect model combined with HR results showed a statistically significant difference between two groups [From time of randomization: HR=0.48 (95% CI: 0.32 to 0.72), Z=3.55, P<0.001; From time of tissue procurement: HR=0.51 (95% CI: 0.33 to 0.78), Z=3.07, P=0.002], implying that Gemogenovatucel-T (Vigil) immunotherapy has better OS in advanced OC patients than placebo (**Figure 2**).

#### 3.4.2 Recurrence-free survival

**Figure 3:**
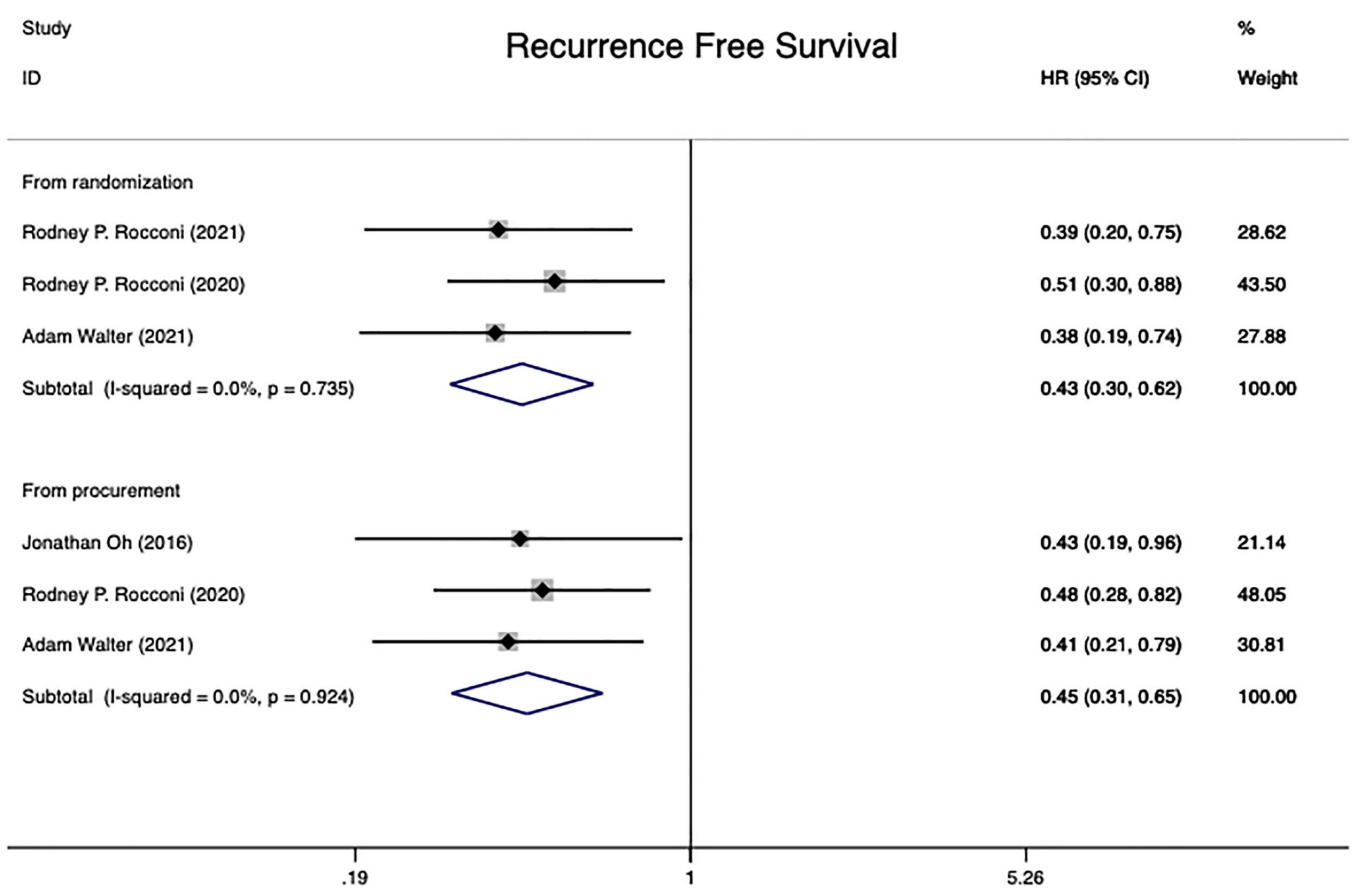
Recurrence free survival in advanced OC patients at randomization and from time of tissue procurement time by using a fixed-effect model. OC, ovarian carcinoma; CI, confidence interval; HR, hazard ratio.

Four independent randomized controlled trials (15, 20, 21, 28) involving 223 patients were included in this analysis. The RFS for 181 patients was conducted from time of randomization, while 178 patients were analyzed from the date of tissue procurement. The analysis of heterogeneity showed that there was no heterogeneity in either group (From time of randomization: I^2 =^ 0.0%, P=0.735; From time of tissue procurement: I^2 =^ 0.0%, P=0.924). The pooled HR from randomization and time of tissue procurement revealed statistical significance in two groups at RFS in advanced OC patients [from randomization: HR=0.43 (95% CI: 0.30 to 0.62), Z=4.61, P0.001; from time of tissue procurement: HR=0.45 (95% CI: 0.31 to 0.65), Z=4.26, P0.001]. The conclusion could be taken that Gemogenovatucel-T (Vigil) immunotherapy had a better RFS than placebo in advanced OC (**Figure 3**).

#### 3.4.3 Recurrence-free survival median time

**Figure 4:**
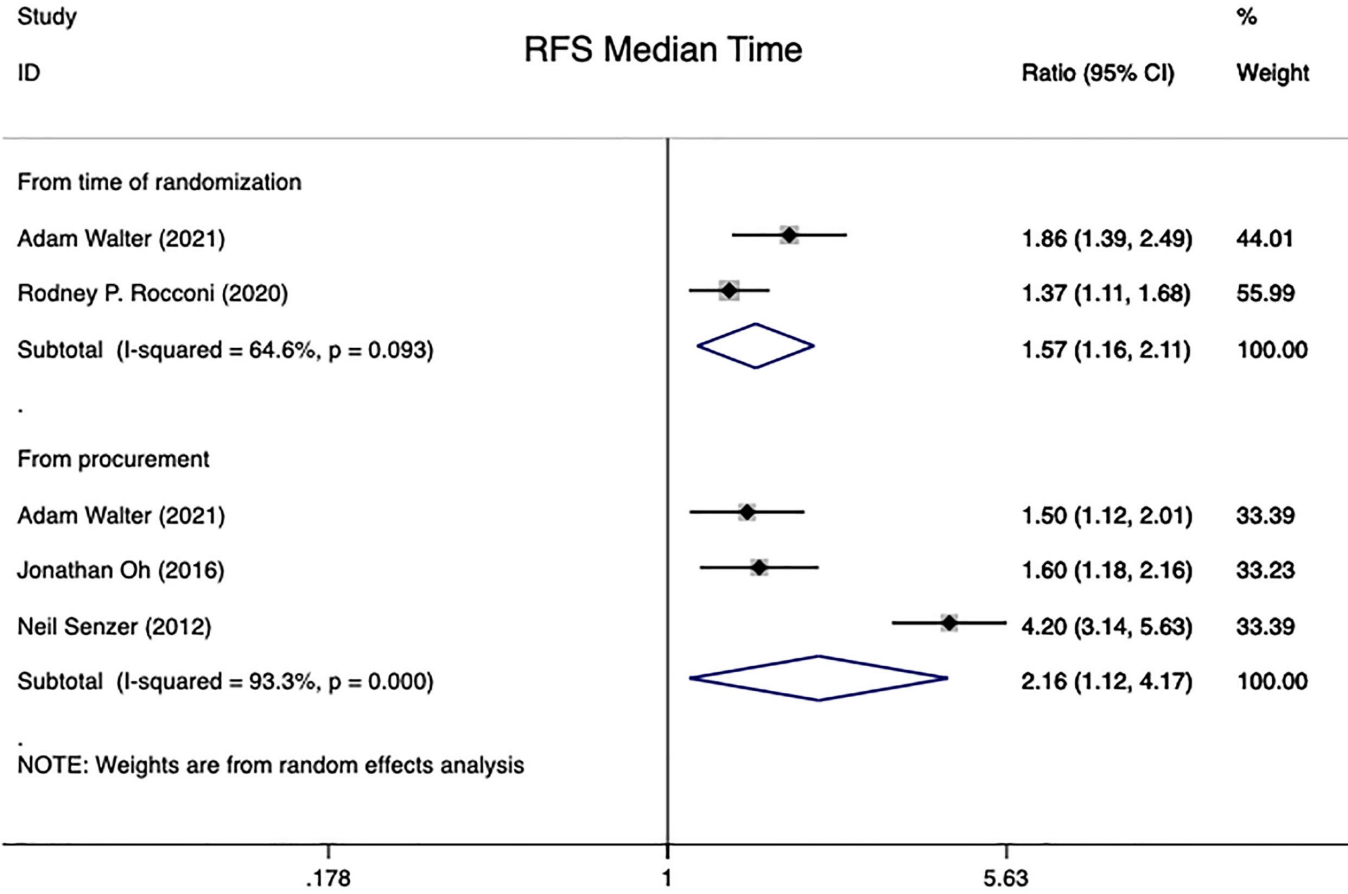
RFS median time in advanced OC patients at randomization and from time of tissue procurement time by using a random-effect model. OC, ovarian carcinoma; CI, confidence interval; HR, hazard ratio; RFS, recurrence free survival.

In summary, the results of four studies could be aggregated into this analysis (15, 17, 20, 28). 268 patients with advanced ovarian carcinoma were included. The RFS median time for 136 of these patients was calculated from the time of randomization, whereas the analysis for 132 patients were performed from the time of tissue procurement. Pooled HR on advanced OC patients showed that Gemogenovatucel-T (Vigil) immunotherapy, compared to Placebo, significantly improved patients’ RFS median time using a random effects model [From time of randomization: HR=1.57 (95% CI: 1.16 to 2.11), Z=2.95, P=0.003; From time of tissue procurement: HR=2.16 (95% CI: 1.12 to 4.17), Z=2.29, P=0.022]. Meanwhile, heterogeneity was high in both groups of RFS median time studies (From the time of randomization: I^2 =^ 64.6%, P=0.093; From time of tissue procurement: I^2 =^ 93.3%, P<0.001) (see in **Figure 4**).

### 3.5 Gemogenovatucel-T (Vigil) related complications

Outcomes for patients who received Gemogenovatucel-T (Vigil) with severe toxicity features (G3/G4) were also pooled and analyzed for all included studies. According to the results, there were no grade 3, 4, or 5 adverse events related with Gemogenovatucel-T (Vigil) described in six publications (2, 15, 20, 21, 25, 28). Only one literature (17) reported the observation of two potentially relevant grade 3 adverse events: abdominal pain and neutropenia.

## 4 Discussion

The purpose of the study, which is the first meta-analysis based on randomized controlled trials (RCTs), is to investigate at the efficacy and safety of Gemogenovatucel-T (Vigil) immunotherapy for advanced ovarian carcinoma. We summarized overall survival (OS), recurrence free survival (RFS), recurrence free survival median time (RFS median time), and complications from seven randomized controlled studies. Gemogenovatucel-T (Vigil) accounts for a significant improvement in terms of OS, RFS, and RFS median time in advanced ovarian carcinoma, as determined by the results of this meta-analysis. Furthermore, as compared to another therapy (placebo), there were mostly no grade 3, 4 or 5 adverse events associated with Gemogenovatucel-T (Vigil). Only one literature (17) reported two potentially relevant grade 3 adverse events (abdominal pain and neutropenia).

In clinical practice, the primary treatment is currently debulking surgery followed by platinum-based chemotherapy (29). For more than two decades, the standard chemotherapy for primary OC has been single-agent chemotherapy or platinum doublet regimens (30). While various attempts have been undertaken, such as maintenance or combination therapy, they have not been very effective in improving the prognosis of patients with advanced ovarian carcinoma. The development of PARPi, on the other hand, has offered another promising therapy option for advanced OC patients. Previous research found that BRCA-mutated OC patients, regardless of gender or medical status, were more likely to benefit from PARPi (Olaparib) (31). Further confirmation from the SOLO1 (9) and SOLO2 (32) trials, OC patients with BRCA mutations have achieved a durable survival benefit from PARP inhibitors. Although much of literature reports a large proportion of the benefits of PARPi observed in OC patients with BRCA-m or BRCA-wt HRD tumors (9, 10, 12), some investigations suggest that angiogenesis inhibitors or PARPi do not indicate an OS benefit in BRCA-wt HRP patients (13). Meantime, among all PARP inhibitors, some studies have shown a worryingly high rate of grades level three or four substance-related adverse interaction and toxicity-related dose interruptions, which mainly includes the hematological and gastrointestinal system (12, 33, 34). Gemogenovatucel-T is an autologous tumor cell vaccine, made from harvested tumor tissue, and the drug has only been used in the treatment of ovarian carcinoma in published studies. However, there is a lack of comparison with the efficacy of other conventional treatment modalities. A randomized crossover design (NCT03073525) comparing Vigil and Vigil + Atezolizumab, which is expected to be completed in September 2022 and deserves our attention. In this meta-analysis, Gemogenovatucel-T treatment has a good safety profile, with only one publication reporting grade 3 adverse effects (17). Additionally, the bulk of the benefits of Gemogenovatucel-T were due to the BRCA-wt group, most of whom are sufferers of homologous recombinant skilled (HRP), meaning that further evaluations of Gemogenovatucel-T may involve patient groups that do not respond well to PARP inhibitors (35).

Several limitations of this meta-analysis could be outlined as follows. First, although we conducted a comprehensive search of mainstream databases, this meta-analysis still had too few studies to include, and only a small number of patients were involved, which may have posed some limitations for clinical reference. Second, due to the small amount of literature included, we did not analyze it for publication bias. Third, the participants were all from the United States, which does not explain the potential of the Gemogenovatucel-T (Vigil) immunotherapy to adapt in various contexts and ethnicities. Nevertheless, it should be noted that this meta-analysis had many advantages. This is the first meta-analysis to explore whether Gemogenovatucel-T (Vigil) immunotherapy has an obvious advantage over other therapies based on randomized controlled trials (RCTs), which provides evidence-based medical evidence on Gemogenovatucel-T (Vigil) treatment selection in the clinical setting. Furthermore, this study demonstrated a clear beneficial effect in women with BRCA wild-type tumors, especially in women with BRCA-wt HRP advanced OC. Moreover, the excellent quality of the literature we eventually included contributes to the credibility of our meta-analysis.

Although immune checkpoint inhibitors (atezolizumab, avelumab, etc.) are now the mainstay of treatment for patients with advanced ovarian carcinoma, majority of clinical trials with checkpoint inhibitors failed in ovarian cancer. The Gemogenovatucel-T (Vigil) immunotherapy has shown to be superior, thus more research is expected in the future on whether this treatment will be effective for patients with advanced ovarian carcinoma.

## 5 Conclusion

This systematic review and meta-analysis based on seven RCTs revealed significant OS and RFS benefits with the Gemogenovatucel-T (Vigil) immunotherapy, particularly in advanced OC patients with BRCA wild type. At the same time, treatment with the Gemogenovatucel-T (Vigil) is safer than other treatment modalities and is not associated with toxic effects in our research. However, the result was based on a small number of studies, including only patients from the United States. As a result, we anticipate further multi-center clinical trials in the future to investigate the efficacy and safety of Gemogenovatucel-T (Vigil) immunotherapy for advanced ovarian carcinoma in a variety of settings and ethnic populations.

## Data availability statement

The original contributions presented in the study are included in the article/**Supplementary Material**. Further inquiries can be directed to the corresponding author.

## Author contributions

YXZ: Document Retrieval, Data Extraction, Data analysis and Essay writing. YLZ and SW: Data Extraction and Data analysis. LZ: Article innovation. LF: Article innovation and Paper submission. All authors contributed to the article and approved the submitted version.

## Funding

This work was funded by the National Natural Science Foundation of China (31900889).

## Conflict of interest

The authors declare that the research was conducted in the absence of any commercial or financial relationships that could be construed as a potential conflict of interest.

## Publisher’s note

All claims expressed in this article are solely those of the authors and do not necessarily represent those of their affiliated organizations, or those of the publisher, the editors and the reviewers. Any product that may be evaluated in this article, or claim that may be made by its manufacturer, is not guaranteed or endorsed by the publisher.

## Abbreviations

OC, ovarian carcinoma; BRCA-wt, BRCA wide type; PARPi, poly (ADP-ribose) polymerase inhibitor; HRD, homologous recombination deficiency; HRP, homologous recombination proficient; RCTs, randomized controlled trials; ROC, receiver operating characteristic curve; AUC, area under the curve; CI, confidence interval; OS, overall survival; RFS, recurrence free survival.
