# Supplementary material for: Efficacy and safety of Gemogenovatucel-T (Vigil) immunotherapy for advanced ovarian carcinoma: A systematic review and meta-analysis of randomized controlled trials
Source: Front Oncol. 2022 Oct 21;12:945867. doi: 10.3389/fonc.2022.945867 (PMC9634109; doi:10.3389/fonc.2022.945867)
Supplement: Supplementary file 1 [file DataSheet_1.docx]

**Supplementary materials**

**Supplementary materials caption**

**Figure S1.** Risk of bias graph for each included study.

**Figure S2.** Risk of bias summary for each included study.

**Supplementary Table S1** General features of the included literature

**Supplementary Table S2** Search process in PubMed

**Supplementary Table S3** Search process in Web of Science

**Supplementary Table S4** Search process in Embase

**Supplementary Table S5** Search process in Cochrane Library

**Figure S1.** Risk of bias graph for each included study.


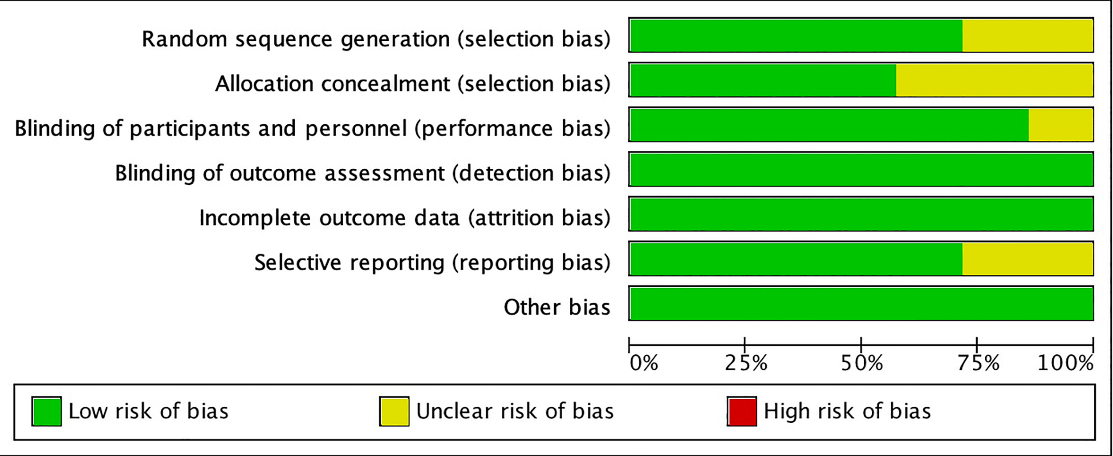


**Figure S2.** Risk of bias summary for each included study.


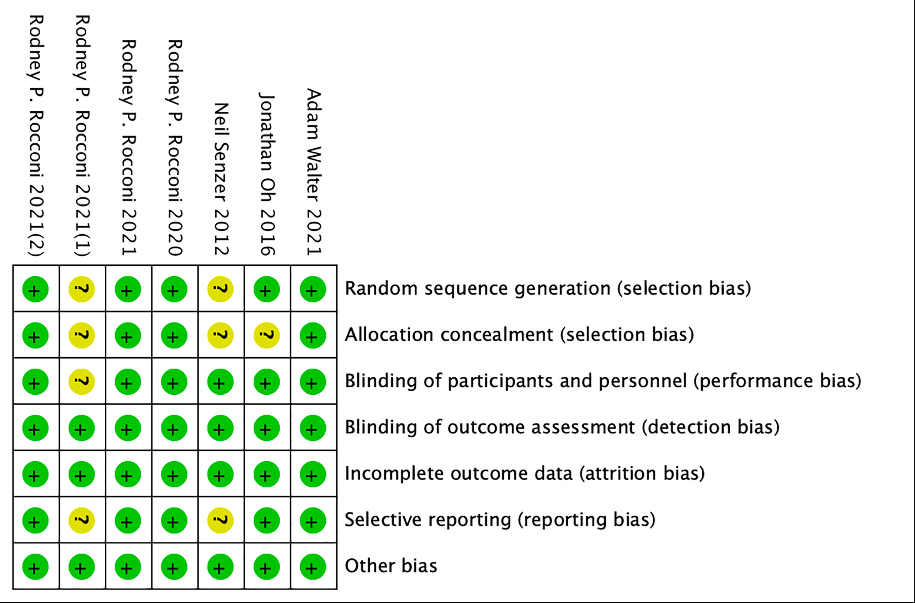


**Supplementary Table S1** General features of the included literature

| References | Year of publication | Frontline chemotherapy | | | | Frontline surgery residual disease | | | | No. of prior lines— no. (%) Median (Range) | |
| --- | --- | --- | --- | --- | --- | --- | --- | --- | --- | --- | --- |
|  |  | T-Neoadjuvant | T-adjuvant | C-Neoadjuvant | C-adjuvant | T-Macroscopic | T-Microscopic/NED | C-Macroscopic | C-Microscopic/NED | Treatment | Control |
| Rodney P. Rocconi | 2021 | 0.83 | 0.17 | 0.841 | 0.159 | 0.32 | 0.68 | 0.3 | 0.7 | NA | NA |
| Rodney P. Rocconi | 2021(1) | NA | NA | NA | NA | 18 (85.7%) | 3 (14.3%) | 10 (83.3%) | 2 (16.7%) | 2 (1-10) | 2 (1-10) |
| Adam Walter | 2021 | 4 (16%) | 21 (84%) | 1 (5.0%) | 19 (95%) | 8 (32%) | 17 (68%) | 6 (30%) | 14 (70%) | NA | NA |
| Jonathan Oh | 2016 | 4 (12.9%) | 27 (87.1%) | 2 (18.2%) | 9 (81.8%) | 11 (35.5%) | 20(64.5%) | 4 (36.4%) | 7 (63.6%) | NA | NA |
| Neil Senzer | 2012 | NA | NA | NA | NA | NA | NA | NA | NA | NA | NA |
| Rodney P. Rocconi | 2020 | 8 (17%) | 39 (83%) | 7 (16%) | 37 (84%) | 16 (34%) | 31 (66%) | 11 (25%) | 33 (75%) | NA | NA |
| Rodney P. Rocconi | 2021(2) | 3 (27.3%) | 8 (72.7%) | 4 (40.0%) | 6 (60.0%) | NA | NA | NA | NA | 2 (1-3) | 2.5 (1-4) |

Note：NA，not available

**Supplementary Table S2** Search process in PubMed

| No. |  | Results |
| --- | --- | --- |
| #1 | "Ovarian Neoplasms"[Mesh] |  |
| #2 | Ovarian Neoplasms [Title/Abstract] |  |
| #3 | Neoplasm, Ovarian [Title/Abstract] |  |
| #4 | Ovarian Neoplasm [Title/Abstract] |  |
| #5 | Ovary Neoplasms [Title/Abstract] |  |
| #6 | Neoplasm, Ovary [Title/Abstract] |  |
| #7 | Neoplasms, Ovary [Title/Abstract] |  |
| #8 | Ovary Neoplasm [Title/Abstract] |  |
| #9 | Neoplasms, Ovarian [Title/Abstract] |  |
| #10 | Ovary Cancer [Title/Abstract] |  |
| #11 | Cancer, Ovary [Title/Abstract] |  |
| #12 | Cancers, Ovary [Title/Abstract] |  |
| #13 | Ovary Cancers [Title/Abstract] |  |
| #14 | Ovarian Cancer [Title/Abstract] |  |
| #15 | Cancer, Ovarian [Title/Abstract] |  |
| #16 | Cancers, Ovarian [Title/Abstract] |  |
| #17 | Ovarian Cancers [Title/Abstract] |  |
| #18 | Cancer of Ovary [Title/Abstract] |  |
| #19 | Cancer of the Ovary [Title/Abstract] |  |
| #20 | #1 OR #2 OR #3 OR #4 OR #5 OR #6 OR #7 OR #8 OR #9 OR #10 OR #11 OR #12 OR #13 OR #14 OR #15 OR #16 OR #17 OR #18 OR #19 | 109183 |
| #21 | Gemogenovatucel-T [Title/Abstract] |  |
| #22 | GMCSF bi-shRNA furin [Title/Abstract] |  |
| #23 | bi-shRNAi (furin) GMCSF DNA autologous tumor cell vaccine [Title/Abstract] |  |
| #24 | Vigil vaccine [Title/Abstract] |  |
| #25 | FANG vaccine [Title/Abstract] |  |
| #26 | #21 OR #22 OR #23 OR #24 OR #25 | 9 |
| #27 | #20 AND #26 | 5 |

**Supplementary Table S3** Search process in Web of Science

| No. |  | Results |
| --- | --- | --- |
| #1 | Ovarian Cancer [Topic] |  |
| #2 | Ovarian Neoplasms [Topic] |  |
| #3 | Neoplasm, Ovarian [Topic] |  |
| #4 | Ovarian Neoplasm [Topic] |  |
| #5 | Ovary Neoplasms [Topic] |  |
| #6 | Neoplasm, Ovary [Topic] |  |
| #7 | Neoplasms, Ovary [Topic] |  |
| #8 | Ovary Neoplasm [Topic] |  |
| #9 | Neoplasms, Ovarian [Topic] |  |
| #10 | Ovary Cancer [Topic] |  |
| #11 | Cancer, Ovary [Topic] |  |
| #12 | Cancers, Ovary [Topic] |  |
| #13 | Ovary Cancers [Topic] |  |
| #14 | Cancer, Ovarian [Topic] |  |
| #15 | Cancers, Ovarian [Topic] |  |
| #16 | Ovarian Cancers [Topic] |  |
| #17 | Cancer of Ovary [Topic] |  |
| #18 | Cancer of the Ovary [Topic] |  |
| #19 | #1 OR #2 OR #3 OR #4 OR #5 OR #6 OR #7 OR #8 OR #9 OR #10 OR #11 OR #12 OR #13 OR #14 OR #15 OR #16 OR #17 OR #18 | 135516 |
| #20 | (FANG vaccine) [Topic] |  |
| #21 | (GMCSF bi-shRNA furin) [Topic] |  |
| #22 | (bi-shRNAi(furin) GMCSF DNA autologous tumor cell vaccine) [Topic] |  |
| #23 | (Vigil vaccine) [Topic] |  |
| #24 | (Gemogenovatucel-T) [Topic] |  |
| #25 | #20 OR #21 OR #22 OR #23 OR #24 | 63 |
| #26 | #19 AND #25 | 7 |

**Supplementary Table S4** Search process in Embase

| No. |  | Results |
| --- | --- | --- |
| #1 | 'ovary cancer'/exp |  |
| #2 | 'ovarian cancer':ab,ti |  |
| #3 | 'ovarian neoplasms':ab,ti |  |
| #4 | 'neoplasm, ovarian':ab,ti |  |
| #5 | 'ovarian neoplasm':ab,ti |  |
| #6 | ovary neoplasms':ab,ti |  |
| #7 | 'neoplasm, ovary':ab,ti |  |
| #8 | 'neoplasms, ovary':ab,ti |  |
| #9 | 'ovary neoplasm':ab,ti |  |
| #10 | 'neoplasms, ovarian':ab,ti |  |
| #11 | 'ovary cancer':ab,ti |  |
| #12 | 'cancer, ovary':ab,ti |  |
| #13 | 'cancers, ovary':ab,ti |  |
| #14 | 'ovary cancers':ab,ti |  |
| #15 | 'ovarian cancer':ab,ti |  |
| #16 | 'cancer, ovarian':ab,ti |  |
| #17 | 'cancers, ovarian':ab,ti |  |
| #18 | 'ovarian cancers':ab,ti |  |
| #19 | 'cancer of ovary':ab,ti |  |
| #20 | 'cancer of the ovary':ab,ti |  |
| #21 | #2 OR #3 OR #4 OR #5 OR #6 OR #7 OR #8 OR #9 OR #10 OR #11 OR #12 OR #13 OR #14 OR #15 OR #16 OR #17 OR #18 OR #19 OR #20 | 93672 |
| #22 | #1 OR #21 | 151737 |
| #23 | 'gemogenovatucel t':ab,ti |  |
| #24 | 'fang vaccine':ab,ti |  |
| #25 | 'bi shrnai':ab,ti AND furin:ab,ti AND 'gmcsf dna autologous tumor cell vaccine':ab,ti |  |
| #26 | vigil vaccine':ab,ti |  |
| #27 | 'gemogenovatucel t':ab,ti |  |
| #28 | #23 OR #24 OR #25 OR #26 OR #27 | 32 |
| #29 | #22 AND #28 | 11 |

**Supplementary Table S5** Search process in Cochrane Library

| No. |  | Results |
| --- | --- | --- |
| #1 | (Ovarian Neoplasms) [MeSH] | 2098 |
| #2 | (Ovarian Neoplasms):ti,ab,kw |  |
| #3 | (Neoplasm, Ovarian):ti,ab,kw |  |
| #4 | (Ovarian Neoplasm):ti,ab,kw |  |
| #5 | (Ovary Neoplasms):ti,ab,kw |  |
| #6 | (Neoplasm, Ovary):ti,ab,kw |  |
| #7 | #2 OR #3 OR #4 OR #5 OR #6 | 3286 |
| #8 | (Neoplasms, Ovary):ti,ab,kw |  |
| #9 | (Ovary Neoplasm):ti,ab,kw |  |
| #10 | (Neoplasms, Ovarian):ti,ab,kw |  |
| #11 | (Ovary Cancer):ti,ab,kw |  |
| #12 | (Cancer, Ovary):ti,ab,kw |  |
| #13 | #8 OR #9 OR #10 OR #11 OR #12 | 5614 |
| #14 | (Cancers, Ovary):ti,ab,kw |  |
| #15 | (Ovary Cancers):ti,ab,kw |  |
| #16 | (Ovarian Cancer):ti,ab,kw |  |
| #17 | (Cancer, Ovarian):ti,ab,kw |  |
| #18 | (Cancers, Ovarian):ti,ab,kw |  |
| #19 | #14 OR #15 OR #16 OR #17 OR #18 | 7460 |
| #20 | (Ovarian Cancers):ti,ab,kw |  |
| #21 | (Cancer of Ovary):ti,ab,kw |  |
| #22 | (Cancer of the Ovary):ti,ab,kw |  |
| #23 | #20 OR #21 OR #22 | 3576 |
| #24 | #1 OR #7 OR #13 OR #19 OR #23 | 8300 |
| #25 | (FANG vaccine):ti,ab,kw |  |
| #26 | (GMCSF bi-shRNA furin):ti,ab,kw |  |
| #27 | (bi-shRNAi(furin) GMCSF DNA autologous tumor cell vaccine):ti,ab,kw |  |
| #28 | (Vigil vaccine):ti,ab,kw |  |
| #29 | (Gemogenovatucel-T):ti,ab,kw |  |
| #30 | #25 OR #26 OR #27 OR #28 OR #29 | 24 |
| #31 | #24 AND #30 | 13 |
